# Autoencoder as a New Method for Maintaining Data Privacy While Analyzing Videos of Patients With Motor Dysfunction: Proof-of-Concept Study

**DOI:** 10.2196/16669

**Published:** 2020-05-08

**Authors:** Marcus D'Souza, Caspar E P Van Munster, Jonas F Dorn, Alexis Dorier, Christian P Kamm, Saskia Steinheimer, Frank Dahlke, Bernard M J Uitdehaag, Ludwig Kappos, Matthew Johnson

**Affiliations:** 1 Neurologic Clinic and Policlinic Departments of Medicine, Biomedicine and Clinical Research University Hospital Basel and University of Basel Basel Switzerland; 2 Department of Neurology Multiple Sclerosis Center Amsterdam Amsterdam University Medical Centers Amsterdam Netherlands; 3 Novartis Pharma AG Basel Switzerland; 4 Neurocenter Luzerner Kantonsspital Luzern Switzerland; 5 Department of Neurology Inselspital University of Bern Bern Switzerland; 6 Microsoft Research Cambridge United Kingdom

**Keywords:** autoencoder, video-rating, machine learning algorithms, deep neuronal network, Neurostatus-EDSS

## Abstract

**Background:**

In chronic neurological diseases, especially in multiple sclerosis (MS), clinical assessment of motor dysfunction is crucial to monitor the disease in patients. Traditional scales are not sensitive enough to detect slight changes. Video recordings of patient performance are more accurate and increase the reliability of severity ratings. When these recordings are automated, quantitative disability assessments by machine learning algorithms can be created. Creation of these algorithms involves non–health care professionals, which is a challenge for maintaining data privacy. However, autoencoders can address this issue.

**Objective:**

The aim of this proof-of-concept study was to test whether coded frame vectors of autoencoders contain relevant information for analyzing videos of the motor performance of patients with MS.

**Methods:**

In this study, 20 pre-rated videos of patients performing the finger-to-nose test were recorded. An autoencoder created encoded frame vectors from the original videos and decoded the videos again. The original and decoded videos were shown to 10 neurologists at an academic MS center in Basel, Switzerland. The neurologists tested whether the 200 videos were human-readable after decoding and rated the severity grade of each original and decoded video according to the Neurostatus-Expanded Disability Status Scale definitions of limb ataxia. Furthermore, the neurologists tested whether ratings were equivalent between the original and decoded videos.

**Results:**

In total, 172 of 200 (86.0%) videos were of sufficient quality to be ratable. The intrarater agreement between the original and decoded videos was 0.317 (Cohen weighted kappa). The average difference in the ratings between the original and decoded videos was 0.26, in which the original videos were rated as more severe. The interrater agreement between the original videos was 0.459 and that between the decoded videos was 0.302. The agreement was higher when no deficits or very severe deficits were present.

**Conclusions:**

The vast majority of videos (172/200, 86.0%) decoded by the autoencoder contained clinically relevant information and had fair intrarater agreement with the original videos. Autoencoders are a potential method for enabling the use of patient videos while preserving data privacy, especially when non–health-care professionals are involved.

## Introduction

In chronic neurological diseases, especially multiple sclerosis (MS), clinical assessment of motor dysfunction is crucial to monitor the disease in patients [1]. Traditional scales used to assess MS, such as the Expanded Disability Status Scale (EDSS), are not sensitive enough to detect slight changes in motor performance [2]. Video recordings of patient performance are more accurate and increase the reliability of severity ratings [3,4]. Moreover, when these recordings are automated, quantitative disability assessments by machine learning algorithms (MLA) can be created [5]. Machine learning algorithms are potentially more sensitive in detecting small changes between images; however, they require high-resolution images because of the high dimensionality of the data [6,7]. Creation of these algorithms usually involves non–health care professionals, which is a potential challenge for maintaining data privacy. Autoencoders can address this issue. They embed visual information into a lower-dimensional latent space that preserves information needed for algorithm development but is not visually interpretable by humans. [6]. An autoencoder consists of an encoder that creates encoded videos by creating a sequence of coded frame vectors and a paired decoder that transforms the coded frame vectors back into the original video. Videos encoded in this way can be shared with non–health care professionals, while the decoder can be used to verify if the essential information from the video has been captured. However, it is unknown whether the condensed data in the coded frame vectors contain clinically relevant data. Therefore, the aim of this proof-of-concept study was to test whether coded frame vectors of autoencoders contain relevant information for analyzing videos of the motor performance of patients with MS.

## Methods

### Study Design and Participants

This study was a subproject of the ASSESS MS study [5] and was approved by the local ethics committees. All participants gave their written informed consent prior to inclusion. In the ASSESS MS study, 9 standardized movements were recorded on video; these movements covered overall motor function, including upper extremity function, truncal stability, and mobility. A detailed description of the movements can be found elsewhere [8]. For this study, we used recordings of the finger-to-nose test. The execution of the finger-to-nose test was standardized using a detailed protocol: Each participant was instructed to close their eyes and abduct their arms to 90° at the shoulder in full extension before touching their nose with the tip of their index finger. Both sides were tested. Original and decoded videos of 20 participants were shown to 10 neurologists at an academic MS center in Basel, Switzerland. The neurologists tested whether these 200 videos in total were human-readable after decoding and rated the severity grade of each original and decoded video according to the Neurostatus-EDSS definitions of limb ataxia [9] (subscore grade 0=no ataxia; grade 1=signs only; grade 2=tremor or clumsy movements easily seen, minor interference with function; grade 3=tremor or clumsy movements that interfere with function in all spheres; and grade 4=most functions are very difficult). The decoded videos were shown firstly, and after an interval of 2-3 weeks, the original videos were shown in the same order to minimize recall bias. The neurologists tested whether these videos were human-readable after decoding.

### Autoencoder

A variational autoencoder was trained on 2230 videos comprising the 9 standardized motor performances included in the ASSESS MS study. The autoencoder was structured so that the frames of each video were encoded into a lower-dimensional space and then decoded into their original form.

**Figure 1:**
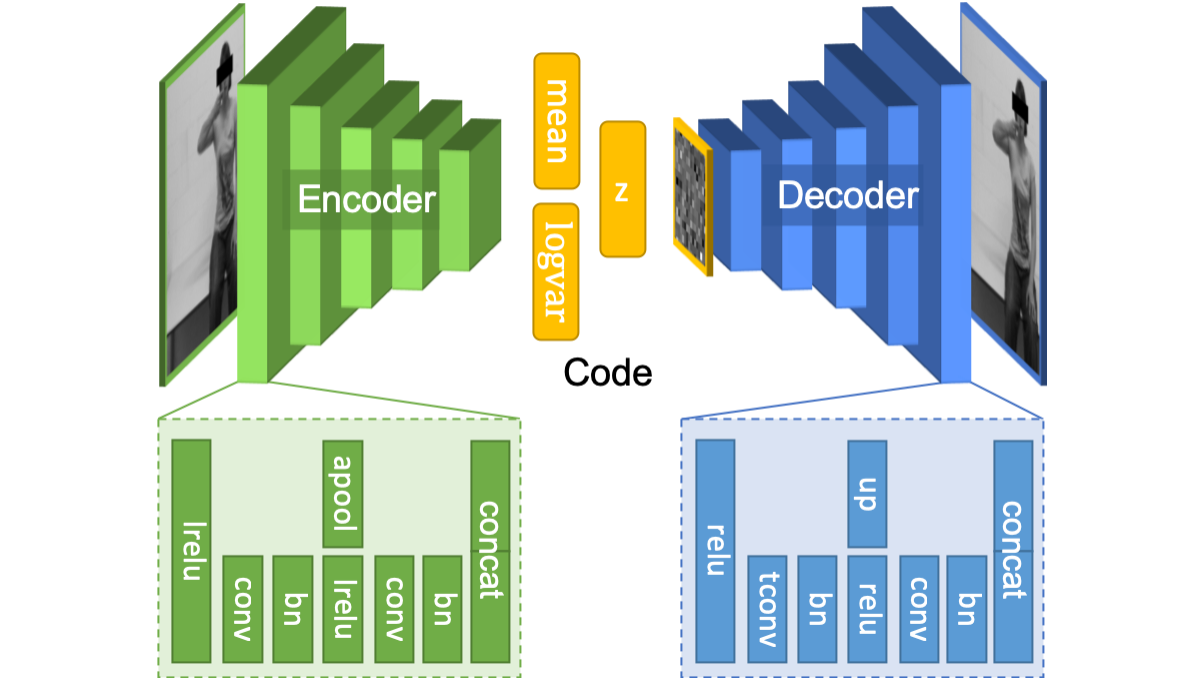
Structure of the variational autoencoder.

Figure 1 depicts the structure of the autoencoder [10]. An encoder network was presented with a single frame from the video without further context. The frame passed through 5 encoding blocks. In each block, the input was processed in a block inspired by a densely connected convolutional network [11], wherein a skip connection was provided between the input and output layers in addition to a convolutional layer/batch normalization sequence. Each block halved the resolution of the image and doubled the feature depth. This network predicted the mean and variance of a normal distribution, which was then sampled to produce a code. The code was presented to a second network that consisted of 5 decoding blocks. Each decoding block consisted of a skip connection (which performed a simple upsampling process) and a transposed convolutional block like that used in a deep convolutional generative adversarial network [12]. Each block doubled the resolution and halved the feature depth. The network was trained using a multi-scale structural similarity–based perceptual loss function [13] with Kullback-Leibler regularization as per Kingma and Welling [10]. The input images were 256×256 RGB-D images with a code length of 256. The training hyperparameters were as follows: the learning rate was 0.001, the convolutional kernel size was 5, and the number of initial filters was 8. The model was trained for 400 epochs.

The key property of interest to us was that when a frame is in its coded form, it is computationally prohibited to decipher it without access to the decoder [6]. An autoencoder as described above reduces the dimensionality of the input data (in our case, videos) by passing the data through an “information bottleneck” [14]. The resulting coded, or latent, space sufficiently describes the data in a way that allows an accurate partial reconstruction. The shared latent embedding is optimized to represent the salient information that is similar across frames of multiple videos (in our case: the movement), whereas dissimilar aspects (eg, background aspects, details of physical features) are less well conserved. Neural networks are a machine learning approach that is inspired by biological neuronal computation; these networks have demonstrated exceptional performance in complex image-related tasks in recent years [15-17]. Given this success, in this study, we used a neural net approach called a variational autoencoder [18]. A variational autoencoder has at its center a coded vector of vastly reduced dimensionality. This is because the decoder requires millions of floating point values to be set precisely before the coded vector can be successfully decoded into an image. At the same time, the coded vector contains all the information necessary to reconstruct that frame; interestingly, due to the variational constraints during training, the frame has semantically meaningful cosine distances to other visually similar frames. This property is very useful for machine learning tasks that operate upon these coded vectors because the coded frames can be used in place of the original video frames without the possibility that a human could use it to recognize the depicted participant.

### Statistics

Intrarater agreement between the ratings of the original and the decoded videos was assessed using the Cohen weighted kappa with linear weights (ie, disagreements of 1, 2, and 3 were weighted by factors of 1, 2, and 3, respectively). A Cohen kappa of 0 corresponds to chance agreement; 0-0.2, to slight agreement; 0.21-0.4, fair agreement; 0.41-0.6, to moderate agreement; 0.61-0.8, to substantial agreement; and 0.81-1, to almost perfect agreement [19]. All analyses were performed in MATLAB (MathWorks, Inc).

## Results

The characteristics of the study population and the participating neurologists are summarized in Table 1.

**Figure 2:**
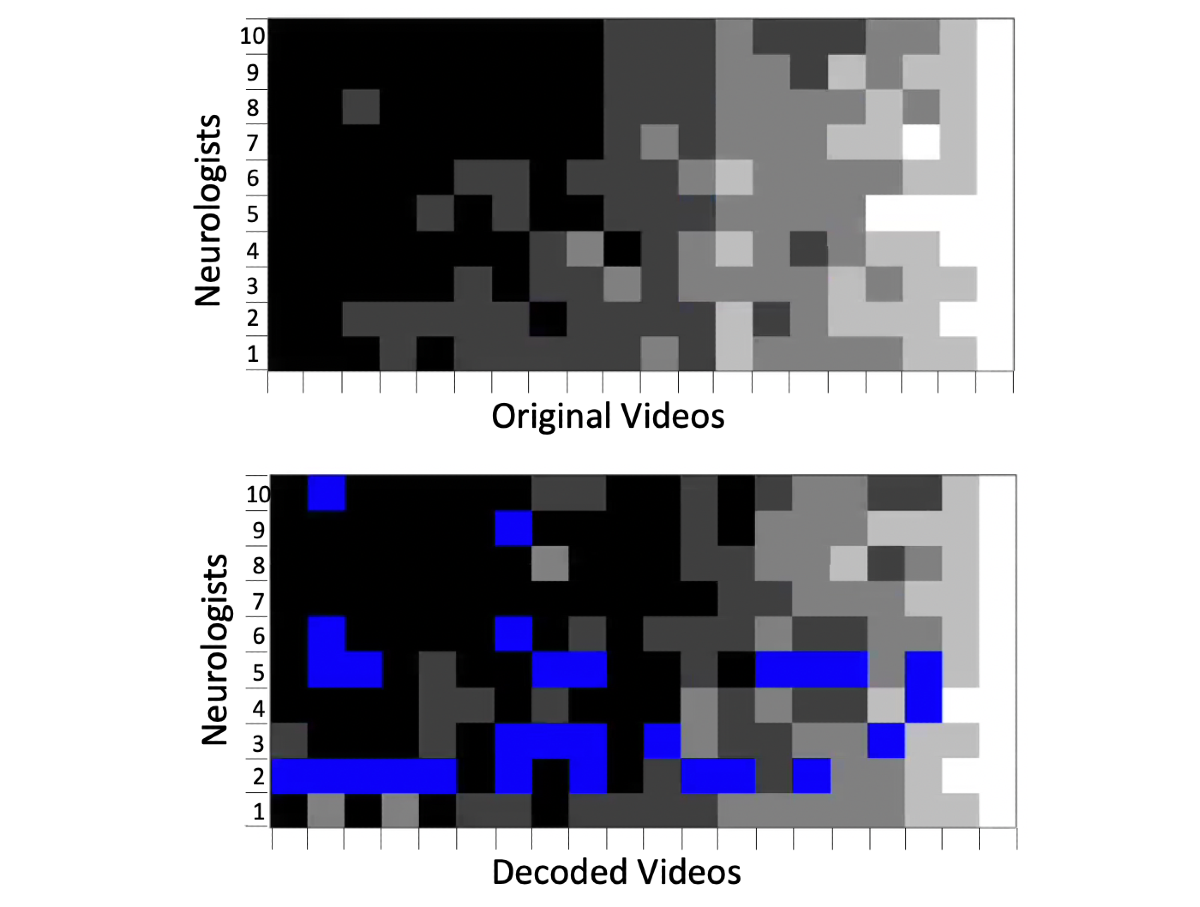
Ratings by 10 neurologists of the original and decoded videos. The colored squares represent the different grades for limb ataxia of the finger-to-nose-test according to the Neurostatus-Expanded Disability Status Scale subscores: black=0, dark grey=1, grey=2, bright grey=3, and white=4. The blue squares represent videos that were judged as not ratable by the neurologists.

In total, 172/200 (86.0%) videos were of sufficient quality to be ratable. The Cohen weighted kappa indicating intra-rater agreement between the original and decoded videos was 0.317. The average difference in the ratings between the original and decoded videos was 0.26, in which the original videos were rated as more severe. The inter-rater agreements of the original and decoded videos were 0.459 and 0.302, respectively. As depicted in Figure 2, agreement was higher when no deficits (grade 0) or very severe deficits (grade 4) were present. Note that most videos that were not ratable were judged so by neurologists 2 and 5.

**Table 1:** Characteristics of the patients and neurologists who participated in the study.

Characteristic			Value
**Patient characteristics (n=20)**			
	Age (years), mean (95% CI)	44.4 (27-74)	
	Gender (female/male), n (%)	12 (63%)/7 (37%)	
	Disease duration (years), mean (95% CI)	13.2 (1-40)	
	Median EDSS^a^ (range)	3.5 (0-6.5)	
	Type of MS^b^ (RRMS^c^/SPMS^d^), n (%)	19 (95%)/1 (5%)	
**Neurologists (n=10)**			
	Gender (female/male), n (%)	5 (50%)/5 (50%)	
	Years of experience in neurology, mean (range)	8.8 (3 to >30)	

^a^EDSS: Expanded Disability Status Scale.

^b^MS: multiple sclerosis.

^c^RRMS: relapsing remitting multiple sclerosis.

^d^SPMS: secondary progressive multiple sclerosis.

## Discussion

### Principal Findings

In this proof-of-concept study, 172/200 (86.0%) of the decoded videos were of sufficient quality to be ratable. We found fair intrarater agreement between the original and decoded videos. The agreement was better for minor and severe deficits in motor function.

Data security and privacy are increasingly requested by health care professionals for data capture, analysis, and storage [20]. At the same time, the use of machine learning algorithms and deep neuronal network techniques as subdomains of artificial intelligence is increasingly infiltrating all areas of health care [21,22]. The use of new technologies and electronic tools for capture and automated analysis of clinical data generally requires the involvement of non–health care professionals, which creates challenges regarding data privacy. To our knowledge, this is the first study to use an autoencoder to allow the analysis of patient videos while preserving data privacy.

Patients with MS may present with slight changes in motor performances over their disease course. Clinical assessment of these changes is notoriously difficult. Video analysis of motor performances allows automated analyses and quantification of disability by using machine learning algorithm–based analysis systems such as those used in the ASSESS MS study; however, it requires a huge data set [5]. Since the creation of machine learning algorithms usually involves non-medical collaborators, encoding of these videos is essential. The intra-rater agreement of original and decoded videos in this study was fair. It is unclear whether this is due to accordance of the video quality or the test-retest reliability of the finger-to-nose test. To our knowledge, no data are available regarding this psychometric property of the finger-to-nose test.

### Limitations

A limitation of this proof-of-concept study is the class imbalance of the patient videos according to the four grades of limb ataxia for the finger-to-nose test [9,21]. Further iterations of the deep neural network are necessary to increase the intrarater reliability.

### Conclusions

In this proof-of-concept study, we have shown that the vast majority (172/200, 86.0%) of videos decoded by an autoencoder contained clinically relevant information regarding upper extremity motor performance represented by the finger-to-nose test and had fair intrarater agreement. Autoencoders are a potential method for enabling the use of patient videos while preserving data privacy, especially when non–health care professionals are involved.

## Abbreviations

EDSS: Expanded Disability Status Scale
MS: multiple sclerosis
RRMS: relapsing remitting multiple sclerosis
SPMS: secondary progressive multiple sclerosis

## References

[1] Cohen JA, Reingold SC, Polman CH, Wolinsky JS (2012). Disability outcome measures in multiple sclerosis clinical trials: current status and future prospects. Lancet Neurol.

[2] van Munster CEP, Uitdehaag BMJ (2017). Outcome Measures in Clinical Trials for Multiple Sclerosis. CNS Drugs.

[3] Burggraaff J, Dorn J, D'Souza M, Morrison C, Kamm CP, Kontschieder P, Tewarie P, Steinheimer S, Sellen A, Dahlke F, Kappos L, Uitdehaag B (2020). Video-Based Pairwise Comparison: Enabling the Development of Automated Rating of Motor Dysfunction in Multiple Sclerosis. Arch Phys Med Rehabil.

[4] D’Souza M, Steinheimer S, Dorn J, Morrison C, Boisvert J, Kravalis K, Burggraaff J, van Munster CE, Diederich M, Sellen A, Kamm CP, Dahlke F, Uitdehaag BM, Kappos L (2018). Reference videos reduce variability of motor dysfunction assessments in multiple sclerosis. Mult Scler J Exp Transl Clin.

[5] Morrison C, D'Souza M, Huckvale K, Dorn JF, Burggraaff J, Kamm CP, Steinheimer SM, Kontschieder P, Criminisi A, Uitdehaag B, Dahlke F, Kappos L, Sellen A (2015). Usability and Acceptability of ASSESS MS: Assessment of Motor Dysfunction in Multiple Sclerosis Using Depth-Sensing Computer Vision. JMIR Hum Factors.

[6] Vieira S, Pinaya WH, Mechelli A (2017). Using deep learning to investigate the neuroimaging correlates of psychiatric and neurological disorders: Methods and applications. Neurosci Biobehav Rev.

[7] Pinaya WHL, Mechelli A, Sato JR (2018). Using deep autoencoders to identify abnormal brain structural patterns in neuropsychiatric disorders: A large‐scale multi‐sample study. Hum Brain Mapp.

[8] van Munster CE, D’Souza M, Steinheimer S, Kamm CP, Burggraaff J, Diederich M, Kravalis K, Dorn J, Walsh L, Dahlke F, Kappos L, Uitdehaag BM (2018). Tasks of activities of daily living (ADL) are more valuable than the classical neurological examination to assess upper extremity function and mobility in multiple sclerosis. Mult Scler.

[9] Kappos, L (2011). https://www.neurostatus.net/.

[10] Kingma DP, Welling M (2019). An Introduction to Variational Autoencoders. Found Trends Mach Learn.

[11] Huang G, Liu Z, Pleiss G, Van Der Maaten L, Weinberger K (2019). Convolutional Networks with Dense Connectivity. IEEE Trans Pattern Anal Mach Intell.

[12] Radford A, Metz L, Chintala S (2016). Unsupervised Representation Learning with Deep Convolutional Generative Adversarial Networks. https://arxiv.org/abs/1511.06434.

[13] Wang Z, Simoncelli EP, Bovik AC (2004). Multiscale structural similarity for image quality assessment. Conference Record of the Thirty-Seventh Asilomar Conference on Signals, Systems and Computers.

[14] Liou C, Huang J, Yang W (2008). Modeling word perception using the Elman network. Neurocomputing.

[15] He Kaiming, Zhang Xiangyu, Ren Shaoqing, Sun Jian (2016). Deep Residual Learning for Image Recognition. IEEE.

[16] Cao Z, Simon T, Wei S, Sheikh Y (2017). Realtime Multi-person 2D Pose Estimation Using Part Affinity Fields. IEEE.

[17] Karras T, Aila T, Laine S, Lehtinen J (2018). Progressive Growing of GANs for Improved Quality, Stability, and Variation.

[18] Kingma D, Welling M (2014). Auto-Encoding Variational Bayes. https://arxiv.org/abs/1312.6114.

[19] Landis JR, Koch GG (1977). The Measurement of Observer Agreement for Categorical Data. Biometrics.

[20] Beinke JH, Fitte C, Teuteberg F (2019). Towards a Stakeholder-Oriented Blockchain-Based Architecture for Electronic Health Records: Design Science Research Study. J Med Internet Res.

[21] Rowe M (2019). An Introduction to Machine Learning for Clinicians. Acad Med.

[22] Triantafyllidis AK, Tsanas A (2019). Applications of Machine Learning in Real-Life Digital Health Interventions: Review of the Literature. J Med Internet Res.

